# Honey Supplementation and Exercise: A Systematic Review

**DOI:** 10.3390/nu11071586

**Published:** 2019-07-12

**Authors:** Samuel P. Hills, Peter Mitchell, Christine Wells, Mark Russell

**Affiliations:** School of Social and Health Sciences, Leeds Trinity University, Horsforth, Leeds LS18 5HD, UK

**Keywords:** carbohydrate, antioxidant, immune function, endurance, intermittent exercise, glucose, fructose

## Abstract

Honey is a natural substance formed primarily of carbohydrates (~80%) which also contains a number of other compounds purported to confer health benefits when consumed. Due to its carbohydrate composition (low glycaemic index, mostly fructose and glucose), honey may theoretically exert positive effects when consumed before, during or after exercise. This review therefore appraised research examining the effects of honey consumption in combination with exercise in humans. Online database (PubMed, MEDLINE, SPORTDiscus) searches were performed, yielding 273 results. Following duplicate removal and application of exclusion criteria, nine articles were reviewed. Large methodological differences existed in terms of exercise stimulus, population, and the nutritional interventions examined. All nine studies reported biochemical variables, with four examining the effects of honey on exercise performance, whilst five described perceptual responses. Acute supplementation around a single exercise session appeared to elicit similar performance, perceptual, and immunological responses compared with other carbohydrate sources, although some performance benefit has been observed relative to carbohydrate-free comparators. When consumed over a number of weeks, honey may dampen immunological perturbations arising from exercise and possibly improve markers of bone formation. More well-controlled research is required to better understand the role for honey in a food-first approach to exercise nutrition.

## 1. Introduction

Honey is defined by European Communities legislation as “the natural sweet substance produced by *Apis mellifera* bees from the nectar of plants or from secretions of living parts of plants or excretions of plant-sucking insects on the living parts of plants, which the bees collect, transform by combining with specific substances of their own, deposit, dehydrate, store and leave in honeycombs to ripen and mature” and is categorised primarily according to origin and mode of production or presentation [1]. A popular foodstuff, honey is comprised of ~80% carbohydrate, and ~19% water [2], and typically contains a wide variety of other components such as organic acids, proteins, amino acids, minerals, polyphenols, vitamins, aroma compounds, and approximately 500 enzymes [2,3]. This diverse profile has seen honey being used for a variety of different health and medicinal purposes [2,3,4,5,6,7]. For example, amongst numerous other proposed health benefits, honey is espoused to have antioxidant, antimicrobial, and anti-inflammatory properties [3,4,5,6,8]. Such effects may be at least partially attributable to honey’s high osmolarity inhibiting bacterial growth, the antimicrobial effects of glucose oxidase and resultant hydrogen peroxide production, and/or the presence of antibacterial substances such as polyphenols [2]. Notably, there exists over 320 different varieties of honey, and the composition of this substance can vary substantially depending upon the variety of plant from which nectar is derived, in addition to the environmental conditions within which the plants grow [2,3,6,9,10]. 

The primary carbohydrates present in honey are the monosaccharides glucose (~30–35%) and fructose (~35–40%). However, ~5–10% of honey’s volume may consist of up to 25 additional di- and trisaccharides in varying quantities [2,3,6,7]. In keeping with the natural variation in composition, the glycaemic index (GI) of honey (and thus potentially the postprandial insulinaemic response) also appears to differ between varieties. Depending upon botanical source, GI values ranging from ~32–85 have been reported [3], with the GI of any given honey appearing to depend largely upon its relative concentration of fructose. Indeed, a higher fructose to glucose ratio is associated with a lower GI value [3,11], and a number of studies have identified fructose-rich varieties of honey, such as Acacia honey, with “low” (i.e., values ≤ 55 [12]) or “moderate” (i.e., values of 55–69 [12]) GI ratings [3,11,12,13].

Carbohydrate has been recognised as an important fuel for exercise since the early 1900s [14], and it is now well established that commencing activity with high concentrations of muscle glycogen may enhance physical performance during exercise of >60–90 min in duration [15,16,17,18]. Moreover, consuming carbohydrates immediately prior to, and during, exercise can help to maintain performance throughout both prolonged endurance events [14,16,19,20,21] and intermittent exercise representative of team sports [14,22,23]. Indeed, carbohydrate ingestion may augment exercise capacity via a sparing of endogenous fuel stores (i.e., muscle and liver glycogen, and blood glucose concentrations), and/or by acting directly on the central nervous system. This latter suggestion is supported by increases in short-term (i.e., events lasting ≤60 min) exercise performance when carbohydrate solutions are simply swilled around the mouth [14,24]. 

The American College of Sports Medicine have provided broad recommendations for carbohydrate consumption during exercise, and therein suggest intakes of ~30–60 g∙h^−1^ [25]. However, whilst it was traditionally believed that ~60 g∙h^−1^ represented the upper limit of carbohydrate (i.e., glucose) oxidation during endurance exercise, more recent evidence suggests that simultaneously ingesting carbohydrates from multiple sources (e.g., glucose and fructose) may increase oxidation capacity by up to ~75% [15,21]. Notably, consuming a combination of glucose and fructose at a rate of 108–144 g∙h^−1^ has improved performance during prolonged cycling exercise when compared with the equivalent dose of glucose alone [14,26,27]. Such developments may be important for athletes engaged in prolonged endurance events (i.e., events of ≥2.5 h in duration) in which fuel availability is likely to be a substantial performance-limiting factor [14]. Given its multiple-carbohydrate composition, there may be a theoretical basis to suggest that honey supplementation could offer a viable and natural alternative to traditional forms of exogenous carbohydrate provision.

In team sports such as soccer, players experience limited opportunities to consume carbohydrates outside of scheduled stoppages in play (i.e., half-time). For this reason, research designs based around a regular feeding pattern throughout exercise (i.e., every 15 min) may be limited in their ecological validity. Moreover, when high GI carbohydrates, including those contained within most commercially available sports drinks, are ingested before and during team sport specific exercise, including at half-time, sharp declines in blood glucose concentrations are typically observed during the early stages of the second half [28,29,30,31]. Given the likely mechanisms involved (for a review of this topic, please see [29]), it has been proposed that altering the GI of carbohydrates consumed pre-match and at half-time, may help to counteract these responses in team sports athletes [29]. Theoretically, low GI carbohydrates produce a lower insulinaemic response and a slower delivery of glucose into the systemic circulation, thus helping to maintain blood glucose concentrations throughout the second half [29,31]. In support, Stevenson et al. [31] observed better maintenance of blood glucose concentrations during the second half of simulated soccer match-play when an 8% solution of low GI isomaltulose (GI: 32) was consumed during the pre-exercise warm-up and at half-time, when compared with an equivalent volume of high GI maltodextrin (GI: 90–100). Whilst no between-trial differences were observed for any performance measure, these findings appear to suggest a potential role for low GI carbohydrate sources for athletes engaged in intermittent team sports when ecologically valid feeding patterns are used.

It is well established that a heavy schedule of prolonged and/or intense exercise can lead to immunity impairment [32,33], and an increased risk of sustaining upper respiratory tract infections [34,35]. An in-depth discussion on the relationship between nutritional strategies and immune responses to exercise is beyond the scope of this article (interested readers please see [33,34,36]), but it is noteworthy that, whilst a viable short term approach for augmentation of endurance training adaptations [15,19,37], exercising in a carbohydrate-depleted state (i.e., following days of low carbohydrate intake and/or prior glycogen depleting exercise) elicits greater elevations in circulating stress hormones and further disruption of several markers of immune function (e.g., interleukin-6; IL-6, interleukin-1 receptor antagonist; IL-1ra and interleukin-10; IL-10), compared with when carbohydrate availability is greater [34]. In addition, consuming ~30–60 g∙h^−1^ of carbohydrates during exercise, particularly throughout prolonged endurance exercise, may also attenuate many of these negative responses through better maintenance of blood glucose concentrations and a concomitant blunting of stress hormone release [33,34,36,38]. 

In addition to carbohydrates, supplementing with antioxidants may have the potential to somewhat counter the immune disturbances experienced following exercise. Whilst evidence for the efficacy of this strategy is at best mixed (see [33]), a 60 day program of antioxidant supplementation attenuated the cytokine (i.e., tumor necrosis factor alpha; TNF-α, interleukin-1 beta; IL-1β, and IL-6) response to a 45 min cycling bout, compared with when the same exercise was completed pre-supplementation [39]. It should be noted that homeostatic disruption may represent a key driver of cellular adaptations to training, and some evidence suggests that interventions aiming to artificially reduce oxidative stress (e.g., supplementing with high doses of antioxidants), have the potential to interfere with molecular signalling [15,33,40]. Although the impact on long-term training adaptations remains unclear, athletes seeking to maintain immune function may wish to initially consider a food-first approach, which prioritises consumption of a range of antioxidant- and phytochemical-rich foods [33].

It has been suggested that honey, due to its high carbohydrate content, may be a suitable energy source for athletes or exercising populations [3]. While possible gastric tolerance issues remain to be confirmed, when consumed around exercise, honey may provide multiple transportable carbohydrates as recommended for endurance athletes, whilst the lower GI of honey compared with that of most commercially available sports drinks has potential applications for athletes engaged in intermittent sports [29,31]. Moreover, honey exhibits natural antioxidant properties that may provide an appropriate balance between controlling the immunosuppressive response to exercise, and maintaining the signalling pathways necessary for positive training adaptation. To this end, a small body of research is beginning to surface surrounding the potential application of honey as a strategy to either enhance athletic performance, improve recovery, or otherwise influence responses to exercise. With this in mind, the aim of the current review was to systematically identify and appraise the current body of research that has examined the effects of honey supplementation in combination with exercise in humans.

## 2. Materials and Methods 

This review was undertaken in accordance with the Preferred Reporting Items for Systematic Reviews and Meta-Analyses (PRISMA) guidelines [41]. Computerised searches were run in the online databases PubMed, Medline, and SPORTDiscus during May 2019, thus articles published up until this time were considered. The search strategy incorporated the terms (honey) AND (exercis* OR soccer OR football OR rugby OR cycling OR resistance-exercis* OR sport* OR dancer OR dancing OR cyclist* OR running OR runner* OR hockey OR basketball OR handball OR swim* OR “team sport*” OR team-sport* OR endurance OR performance OR rowing OR rower* OR sprint OR jump OR power OR strength OR training OR hurling OR weightlift*), and the filters applied were: English language, humans, clinical trial, journal article, and peer-reviewed. References listed within bibliographies of the retrieved records, in addition to articles already known to the authors, were also considered for inclusion. 

### 2.1. Study Selection

Based upon the specific aims of this review, studies identified from the original search strategy were systematically excluded according to the following criteria: (A) studies not conducted with living human participants; (B) studies which involved either no exercise stimulus, no nutritional intervention which included honey, or both; or (C) studies that were review articles. 

### 2.2. Quality Assessment

After application of the pre-defined exclusion criteria, the remaining full text articles were assessed for methodological quality via the Physiotherapy Evidence Database (PEDro) scale. This assessment scores experimental studies out of a maximum of 10 points, based upon satisfaction of a range of criteria. Eight of these criteria evaluated a study’s internal validity, and a further two criteria related to whether sufficient statistical information was presented. The PEDro scale has previously been identified as a valid and reliable indicator of methodological quality [42,43]. As per previous review papers in this field, only articles with a PEDro score of at least five out of 10 were included in order to improve the credibility of the analyses [44].

## 3. Results

**Figure 1:**
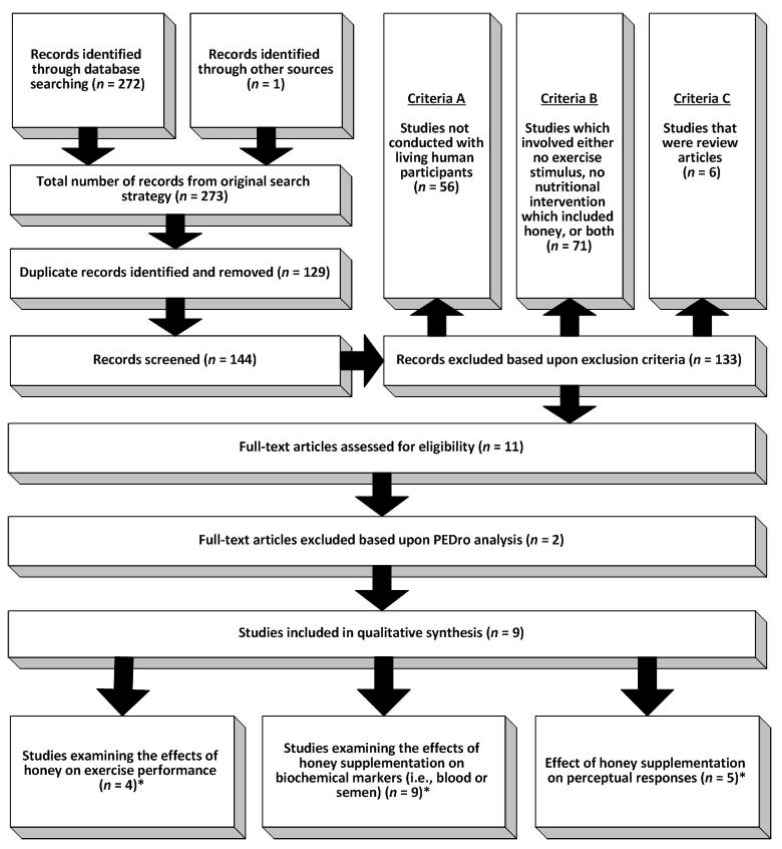
Selection process for articles included in the systematic review. * denotes that some studies examined multiple constructs, thus are included in more than one category.

A total of 273 records were identified through the original search strategy (including one record previously known to the authors). Following removal of 129 duplicates, 144 records were screened according to the pre-defined exclusion criteria. Of the 133 records excluded at this stage, 56 studies were not conducted with living human participants (exclusion criteria A); 71 involved either no exercise stimulus, no nutritional intervention which included honey, or both (exclusion criteria B); whilst six records were excluded on the basis that they were review articles (exclusion criteria C). When the remaining 11 full text articles were assessed for eligibility and quality, a further two records were excluded on the basis of scoring <5 out of 10 on the PEDro scale. Therefore, a total of nine articles were retained and included in this review (Figure 1). 

In the nine eligible articles, outcomes were presented for 186 participants (individual study sample sizes ranging from nine to 40 participants), being mostly amateur athletes, of whom 125 were male and 61 were female. Four studies reported crossover, repeated measures designs, whilst five studies assigned participants to independent groups. Exercise modalities included team sport simulations, running, cycling, rowing, resistance exercise, and dance. Considerable methodological variation also existed with regards to the patterns and dosages of honey supplementation, with some studies feeding honey either before, during or after a single exercise session, and others investigating the effects of honey supplementation over several weeks (e.g., through a training ‘block’). Articles were pooled according to three broad themes and two sub-themes (Table 1, Table 2, Table 3 and Table 4), which are presented in turn below. Studies have examined the effects of honey supplementation (either acutely or over multiple weeks) on (a) biochemical markers, (b) physical and skilled performance, and (c) perceptual responses. A number of articles investigated multiple constructs and were therefore included within more than one theme.

### 3.1. Effect of Honey Supplementation on Biochemical Markers (i.e., Blood or Semen)

All nine eligible studies included at least one outcome variable derived from bodily fluid. Blood samples (venous or capillary) were taken in eight of these instances, whilst the remaining study derived indices of immune status from markers present within semen. As substantial methodological variation was observed, results are separated into (a) studies investigating the acute effects of honey consumption around a single exercise session (Table 1) and (b) those in which honey supplementation occurred over multiple weeks (Table 2).

#### 3.1.1. Acute Honey Consumption around a Single Exercise Session

Five of the nine studies concerned the acute effects of honey supplementation (Table 1). In 59 males and 21 females, honey was administered in various doses, frequencies and forms (i.e., solution, gel, or powder) during rowing [45], cycling [46], and soccer specific exercise [47], as well as between running bouts in hot conditions [48], and immediately following resistance exercise [49]. Partly due to the inherent difficulty in consolidating findings from such different methodological approaches, the influence of acute honey supplementation on blood glucose concentrations and insulin responses during and following exercise remains inconclusive. In addition, consuming honey around a single exercise session appears to produce similar immune and hormonal (e.g., testosterone and cortisol concentrations) responses when compared with consumption of other carbohydrate sources.

#### 3.1.2. Honey Supplementation over Multiple Weeks

The remaining four articles (involving a total of 76 males and 40 females) investigated biochemical changes when honey was consumed over periods ranging from 31 days to 16 weeks (Table 2). Three studies investigated immunological markers, two via blood samples, and one via semen analysis [50,51,52], whilst one study assessed whether honey supplementation combined with aerobic dance exercise influenced markers of bone formation and/or resorption [53]. Consuming 70 g honey prior to each training session over periods of eight to 16 weeks attenuated the negative immune response to a programme of moderate to intense cycling exercise when compared with no nutritional supplement [50,51]. Although potential benefits have been observed, the influence of daily honey supplementation is less clear with respect to markers of bone formation/resorption [53], and when consumed in smaller quantities (i.e., 3 × 10 mL of a honey and yeast product per day) over 31 days of running training [52]. Unfortunately, in each of these four studies it is unclear whether the groups used as comparators were energy or carbohydrate-matched compared with those consuming honey.

### 3.2. Effect of Honey Supplementation on Physical or Skilled Performance

Four eligible studies (Table 3) have assessed the influence of honey supplementation on at least one measure of physical or skilled performance in a total of 53 males. Three studies investigated the acute effects of honey consumption during team sport [47], running [48], or cycling [46] exercise, whilst one study used various cycling ergometer tests to assess the influence of consuming 70 g of honey 90 min prior to each training session on adaptations throughout a 16 week period of training [51]. Whilst benefits were observed when honey was compared to consuming no carbohydrate at all [46,48], findings have been largely inconsistent with regards to the influence of honey supplementation on exercise performance (Table 3).

### 3.3. Effect of Honey Supplementation on Perceptual Responses

Five articles have reported perceptual responses from a total of 59 males and 21 females, when honey was consumed before, during, or immediately after exercise (Table 4). A variety of Likert scales were employed to measure constructs relating to taste, texture, gut comfort, and perceived fatigue [45,46,47,48,49]. Although honey may elicit a sweeter taste compared with water [48], no differences in ratings of perceived exertion or perceptions of fatigue, either during or after exercise, were reported with honey as opposed to water or other forms of carbohydrate.

## 4. Discussion

This review aimed to systematically evaluate the current body of research that has assessed the influence of honey supplementation on a number of physiological, performance, and perceptual responses to exercise. Whilst nine eligible articles were identified, substantial methodological variation exists between studies, thus making it difficult to draw firm conclusions with regards to the potential efficacy of honey supplementation for exercising populations. As is the case with many supplements, it seems likely that a number of factors, including but not limited to, the dose and timing of honey ingestion, individual responsiveness, and the type, duration, and/or intensity of exercise, modulate the responses observed [44].

### 4.1. Effect of Honey Supplementation on Biochemical Markers (i.e., Blood or Semen)

#### 4.1.1. Acute Honey Consumption around a Single Exercise Session

One of the primary aims for athletes consuming carbohydrates before and during exercise is to maintain fuel availability (i.e., preserve glycogen stores and increase blood glucose concentrations) to help attenuate declines in performance as exercise progresses. With regards to honey consumption prior to and/or during an exercise bout, blood glucose responses have been largely inconsistent but notably the effects on glycogen degradation remain to be examined (Table 1).

Only one study has reported a statistically significant difference in blood glucose concentrations as a result of consuming a honey-containing supplement. Lagowska et al. [45] observed lower post-exercise blood glucose concentrations when a “natural” carbohydrate beverage containing honey, fruit juice, and banana was consumed during 80 min (2 × 40 min, separated by 5 min) of rowing exercise, compared with a commercially available carbohydrate solution. Whilst the reasons for these findings remain unclear, the differential responses may be attributable to the amount of carbohydrate consumed. Although both conditions entailed consumption of six boluses of 150 mL of solution (i.e., one every 15 min during exercise), the “natural” drink contained 6.7% carbohydrates, whereas the comparator was a 7.8% solution. It seems likely that differences in the absolute quantity of carbohydrate consumed, irrespective of source, may at least partly explain the differential blood glucose responses in favour of the higher amount. Unfortunately, because the precise quantity of each constituent within the two drinks was not reported, further speculation is rendered difficult.

Blood glucose concentration maintenance may be of particular interest to athletes engaged in sports which require high levels of technical skill (e.g., team sport athletes). Whist a definitive link between hypoglycaemia and reductions in sport-specific physical or skilled performance has not yet been established in exercising participants, the role of blood glucose in brain function is clear. Indeed, the brain is one of the few human organs that relies heavily on blood glucose to maintain optimal functioning [54]. Notably, exercise studies have indicated that the rate of cerebral glucose uptake begins to decline when blood glucose concentrations fall below ~3.6 mmol∙L^−1^ [55], a concentration which has previously been observed in team sport players ~15–30 min following half-time [28]. Moreover, the link between blood glucose concentrations and cognitive functioning is highlighted by increased fine motor speed, psycho-motor speed, and visual discrimination speed accompanying increased glycaemia following soccer match-play in the heat [56]. As cognitive processes are likely to be vital, not only to the skilled actions involved in team sports but also to tactical and/or strategic decision making, nutritional strategies that help maintain or increase blood glucose concentrations could be of benefit to team sports players during the latter stages of a match [29]. Given its typical carbohydrate composition (i.e., containing primarily low GI fructose), there exists a theoretical basis to suggest honey as a potentially worthwhile intervention in this context [29].

When high GI carbohydrates are consumed during team sport specific exercise, a temporary lowering of blood glucose concentrations may be experienced during the early stages of the second half [28,29,30,31]. To date, only one study has combined honey supplementation with simulated soccer match-play, and no differences in insulin or blood glucose concentrations were observed immediately or one hour following exercise cessation when either a honey solution, a carbohydrate-matched commercially available sports drink, or an energy-free placebo were consumed [47]. However, because blood samples were not taken regularly throughout exercise, whether or not honey influenced transient changes in blood glucose responses as seen previously, remains unclear. Given the role of blood glucose concentrations in the maintenance of brain function, future research into the potential influence of honey supplementation on blood glucose concentrations, in addition to physical, skilled, and cognitive performance throughout intermittent exercise, would be worthwhile.

Other studies involving honey consumption prior to and/or during exercise have also reported no significant treatment effects for blood glucose concentrations [46,48]. Notwithstanding, blood glucose concentrations were maintained throughout a 64 km cycling time-trial when 15 g of either honey or dextrose were consumed every 16 km [46]. In contrast, in the placebo condition, blood glucose concentrations had declined after 48 km relative to the initial 16 km [46]. Although no differences between honey and dextrose were observed, it is unclear whether the honey and dextrose trials were carbohydrate- or energy-matched. Indeed, whilst the authors state that 15 g of gel was consumed in each condition, it should be considered that whereas dextrose is ~100% carbohydrate, honey in its natural form may be only ~80% carbohydrate or less [2]. The lack of information regarding whether carbohydrates were consumed in equivalent doses makes interpretation of the results difficult, but if discrepancies existed, the differences in absolute carbohydrate intake may have influenced the results in addition to the source of carbohydrates alone.

Alongside providing amino acids for muscle repair and fluid for rehydration, rapid replenishment of glycogen stores is of primary importance for facilitating recovery following exercise [17,57]. Moreover, co-ingesting carbohydrates and protein during the post-exercise period may help to promote an anabolic environment, offset the acute immunosuppressive effects of intense exercise, and facilitate glycogen restoration [17,19,33,57]. Krieder et al. [49] investigated the effects of consuming 40 g whey protein alongside 120 g of either sucrose, powdered honey, or maltodextrin within five minutes of completing a bout of resistance exercise. When compared with individuals taking no supplement, all three carbohydrate groups returned higher insulin concentrations at 30, 60, 90 and 120 min post-feeding. Although this may be expected given that carbohydrates and whey protein are insulinogenic [58], it is interesting to note that blood glucose concentrations in the honey group were higher after 30 min post-feeding compared with those in the sucrose group, and higher than all three other conditions at 60 min following consumption. In keeping with the lower GI of honey compared with the other sources of carbohydrate assessed, these patterns appear to suggest a more prolonged appearance of carbohydrate in the bloodstream when honey was consumed. Whilst no treatment effect was observed for testosterone or cortisol, these blood glucose and insulin responses may suggest potential for the use of honey when post-exercise recovery is required.

When honey is consumed prior to, and/or during, a single exercise session, limited effects have been observed with regards to immunological markers (Table 1). Although the plasma IL-1ra response to team sport specific exercise was dampened following honey supplementation (i.e., a 6% solution providing a total of 1 g∙kg^−1^ body mass) compared with when a commercial sports drink or carbohydrate-free placebo were consumed, similar IL-6 responses were observed [47]. The authors thus proposed that the antioxidant and/or polyphenol content of the honey supplement may have somewhat interrupted the intracellular production or release of IL-1ra in response to exercise-induced elevations in IL-6. Conversely, previous carbohydrate research showed that ingesting a 6.4% glucose and maltodextrin solution before, and at 15 min intervals during, simulated soccer match-play attenuated a number of post-exercise immune disturbances (i.e., increases in plasma cortisol and IL-6, and inhibition of bacterially stimulated neutrophil degranulation), compared with a placebo [59]. Although comparisons are complicated by inconsistencies in the specific methods employed (i.e., length of exercise bout, pattern, dose, and type of carbohydrate provision, and the time-frame of measurement), it appears plausible that differences in blood glucose responses may at least partially explain the divergent findings. In support, increases in stress hormone concentrations during exercise may be attenuated when blood glucose is maintained via the feeding of exogenous carbohydrates [33,34,36,38]. That said, Abbey and Rankin [47] observed no differences in blood glucose concentrations between the honey, commercial beverage, and placebo conditions.

Although the evidence remains limited (Table 1), other studies have also shown that honey exhibits similar effects on immunological responses compared with other carbohydrate sources, at least where acute consumption around a single exercise session is concerned [45,49]. More well-controlled research is therefore required to determine whether honey has any ameliorating influence on immune markers, compared with that provided by other sources of carbohydrate.

#### 4.1.2. Honey Supplementation over Multiple Weeks

In contrast to findings from acute honey supplementation studies, the literature available to date suggests that honey, consumed over the course of multiple weeks, may dampen the inflammatory response to periods of repeated exercise [50,51]. Indeed, ingesting 70 g of unprocessed honey ~90 min prior to each training session over 16 weeks of moderate-to-intense cycling training resulted in reduced plasma cytokine (i.e., IL-1β, IL-6, IL-10, and TNF-α) concentrations immediately, 12 h, and 24 h after the last training session in the eighth week, and immediately, 12 h, 24 h, and seven days after the last training session in week 16, compared with when no supplement was consumed [51]. Moreover, glutathione levels and total antioxidant status were higher with honey supplementation. Very similar patterns of cytokine and antioxidant responses were reported when Tartiban et al. [50] implemented the same supplementation and exercise strategy over an eight week period. This latter study, albeit investigating semen as opposed to blood markers, also noted reductions in indices of oxidative stress (i.e., reactive oxygen species and malondialdehyde) when honey was consumed throughout the eight week period. Whilst oxidative stress may play an important role in cellular adaptations to training [15,33,40], these studies in amateur male cyclists appear to demonstrate a potential application for honey supplementation during periods in which reductions in exercise-induced immune disturbances are desired. However, whilst it was stated that all participants were advised to maintain their normal diets for the duration of both investigations, and that diet diaries were completed to ensure compliance, neither articles provided detailed information outlining participants’ overall energy, or macronutrient intake and/or distribution. As carbohydrate availability may influence immunological responses to exercise [34], it remains unclear whether the reported results stem from the inherent properties of honey itself, or simply reflect the outcome of an increased carbohydrate or energy intake in the honey group. In addition, more research, using ecologically valid and robust methods, is required to determine whether attenuation of acute immunological perturbations results in decreased incidences of infection.

In contrast to the above studies in cyclists [50,51], Gmunder et al. [52] reported no significant between-group differences in immunological responses to a 21 km run completed after 27 days of treatment when participants consumed 30 mL per day of either a supplement containing (a) herbal yeast, malt, honey, and orange juice, or (b) sucrose and caramel. Whilst definitive conclusions are difficult to draw, it seems plausible that the absolute quantity of honey consumed by runners in this study was insufficient to confer any antioxidant effect. Indeed, the primary health benefits of honey consumption have been demonstrated with intakes >50 g [3]. Considering the paucity of well-controlled studies published to date, further work is required to investigate the immunological effects of acute and longer term honey supplementation within exercising populations. In particular, research examining the effects of different variables which may influence the efficacy of supplementation; such as dose, duration and/or form of honey supplementation, different exercise modalities and/or intensities, different levels of athlete, and/or the role played by an individual’s starting nutritional status, would be of particular interest [3,44]. From a practical perspective, the relationship between potential reductions in immunological perturbations as a result of honey supplementation and (a) the risk of developing an infection, (b) recovery and subsequent exercise performance, and (c) long-term training adaptations, must represent a research priority to help guide practitioners and athletes.

Ooi et al. [53] studied 37 females over the course of six weeks with an interest in markers of bone formation (i.e., serum alkaline phosphatase) and resorption (i.e., serum C-terminal telopeptide of type 1 collagen). Participants were assigned to one of four groups, whereby two groups performed aerobic dance exercises three times per week (with one group also consuming 20 g honey per day), whilst the remaining two groups remained sedentary (again with one group also consuming 20 g honey per day). Although there were no differences for either outcome variable when groups were directly compared, the two groups who consumed honey experienced significant increases in serum alkaline phosphatase, tentatively indicating greater bone formation over the six week period. In contrast, both non-supplemented groups maintained similar levels at week six compared with week zero [53]. Honey supplementation has previously shown potential to enhance markers of bone structure in rodent studies [60], and it could be the case that certain components such as vitamin K, and minerals such as calcium, phosphorus, iron, and magnesium found in honey contribute to improved bone health, especially when combined with an exercise programme.

### 4.2. Effect of Honey Supplementation on Physical or Skilled Performance

It is recommended that consuming carbohydrates prior to, and during, exercise may enhance indices of physical (i.e., time to exhaustion) and skilled (i.e., soccer passing and shooting accuracy) performance [14,16,19,20,21,22,23,44]. Moreover, combining different forms of carbohydrates may increase oxidation rates and allow for worthwhile ingestion of a greater overall total volume of exogenous energy during prolonged endurance exercise [14,26,27]. Given that honey contains multiple sources of carbohydrates (i.e., primarily fructose and glucose), this natural substance seems intuitively able to offer potential as a “food-first” approach to carbohydrate supplementation. However, studies investigating the influence of honey on physical or skilled performance have reported mixed results (Table 3), with only one investigation reporting a clear performance benefit [48].

When amateur runners used a 6.8% Acacia honey solution to restore ~150% of body mass losses following a 60 min run in the heat, improved running performance (i.e., distance covered) versus ingestion of an equivalent volume of water occurred during the 20 min treadmill test that followed 120 min later [48]. Given the established role of carbohydrate–electrolyte beverages in fuelling for, and recovering from, exercise, such findings are not unexpected. However, because no carbohydrate-matched alternative to honey was assessed, it cannot be determined whether the positive outcomes are linked the unique properties of honey, or a result of carbohydrate consumption (i.e., as opposed to water) per se.

Whilst no significant between-condition differences were observed for overall time taken to complete a 64 km time-trial, ingesting 15 g of either honey or dextrose in gel form every 16 km enabled cyclists to maintain average 16 km time throughout the duration of exercise, and to increase average power output during the final 16 km compared with the preceding 16 km segments [46]. In contrast, when an energy-free placebo was consumed, significant declines in performance were observed over 48–64 km compared with the opening 16 km of exercise. Although this investigation identified no differences between honey and dextrose for any performance measure, the rate of carbohydrate consumption may have been a factor. Indeed, because carbohydrates consumed from a single source (i.e., glucose) may be oxidised at up to ~60 g∙h^−1^, the full benefits of consuming multiple transportable carbohydrates (i.e., in terms of increased oxidation rates) may not be realised until absolute carbohydrate intake exceeds this level [14]. Moreover, a clear dose-response relationship exists between carbohydrate intake and endurance performance [14,61,62]. As noted above in relation to blood glucose responses, it is unclear whether the honey and dextrose trials were carbohydrate- or energy-matched. Due to reporting being vague on this matter, there is the potential that discrepancies in absolute carbohydrate intake may have influenced the results in addition to the source of carbohydrates alone.

With regards to intermittent exercise, carbohydrate supplementation has previously demonstrated benefits in terms of maintaining physical and skilled performance during the latter stages of simulated team sport match-play, whether consumed frequently (i.e., every 15 min) during exercise [28,30] or in more ecologically valid feeding patterns (i.e., before exercise and at half-time) [23]. However, ingesting a honey solution containing 6% carbohydrates demonstrated no benefit to any measure of physical or skilled performance assessed either during, or immediately following, 75 min of soccer specific exercise, when compared with a carbohydrate-matched commercially available sports drink and an energy-free placebo [46]. Whilst it may appear surprising that neither carbohydrate intervention influenced performance, it has been suggested that when limited opportunities exist to ingest carbohydrates, consuming solutions containing upwards of 10% carbohydrates may enable ergogenic rates of energy intake (e.g., >50 g∙h^−1^) to be achieved whilst minimising abdominal discomfort [29]. For example, Harper et al. [23] observed significant improvements in dribbling speed and self-paced exercise performance during the latter stages of simulated soccer match-play when a 12% carbohydrate–electrolyte solution was delivered in 250 mL boluses prior to the beginning of each half, compared with either water or an electrolyte placebo. Future research into the effects of consuming higher concentrations of honey (i.e., ≥10% carbohydrate) on physical, skilled, and cognitive performance during team sport specific exercise would be beneficial.

Whilst short periods of deliberately training with low endogenous and exogenous carbohydrate availability may promote positive training adaptations through increases in mitochondrial enzyme activity, increases in lipid oxidation, and potential improvements in exercise capacity [15,19,37], consuming a diet rich in carbohydrates may help athletes to fuel and recover from training and thus itself promote beneficial responses via an increase in training intensity [19]. In the only eligible study to have investigated the performance effects of honey supplementation over a period of multiple days, amateur male road cyclists who supplemented with 70 g of honey 90 min prior to each training session demonstrated no additional improvements in 5 km or 40 km cycling time-trial performance, or peak power output over a 16 week period compared with those taking no supplement [51]. Unfortunately, detailed dietary analysis was not provided, thus it is not possible to comment upon the adequacy of, or differences in, overall carbohydrate intake in either group over the 16 week duration. Well-controlled research to determine whether longer-term honey consumption translates into favourable performance adaptations will be difficult to conduct. Whilst longer-term consumption may have a number of other benefits for health and wellbeing [3,5,7], research into the performance effects of honey supplementation on an acute level (i.e., immediately prior to and/or during exercise) may have a more sound theoretical underpinning.

### 4.3. Effect of Honey Supplementation on Perceptual Responses

Honey has demonstrated similar effects on subjective ratings of effort, compared with when other sources of carbohydrates, energy free placebos, or water have been consumed (Table 4). Such findings reflect previous reports in relation to intermittent exercise, whereby nutritional interventions failed to influence perceived exertion [30,31]. Moreover, whilst possible reductions in ratings of perceived exertion have been observed during prolonged endurance cycling when a mixture of glucose and fructose were consumed versus glucose alone, these responses occurred at substantially higher rates of carbohydrate ingestion (i.e., 90 g∙h^−1^) than those provided by the studies presented in this review [63]. Indeed, the only perceptual responses affected by honey consumption relate to flavour and texture as opposed to exertion or fatigue. Specifically, a honey solution elicited a sweeter taste compared with plain water [48], and a natural beverage based upon honey, banana, and fruit juice provided a less satisfying consistency than a commercially available sports drink [45]. Within tolerable limits, sweeter tasting beverages may promote increases in voluntary fluid intake during exercise [64], and such findings may suggest a potential application of honey to encourage a greater rate of carbohydrate consumption in individuals suffering from flavour fatigue.

The fact that a honey solution (6% carbohydrate) produced similar perceptions of thirst, nausea, fullness, and stomach upset compared with plain water [48], may be an important observation. One of the traditional concerns with recommending low GI carbohydrates around exercise has surrounded the risk of gastric distress when the time-frame for appearance of exogenous carbohydrate is prolonged [65]. Although Ahmad et al. [48] only assessed perceptual responses immediately after each carbohydrate feeding, and not during the 20 min exercise bout that followed, previous research incorporating soccer match simulation has shown similar abdominal discomfort values when an 8% isomaltulose solution was consumed, compared with an equivalent volume of high GI maltodextrin [31]. Taken together, these studies may provide food for thought for practitioners and athletes who may previously have been deterred from considering lower GI carbohydrates before and during exercise.

## 5. Conclusions and Future Research Recommendations

Due to the potential health benefits and to offset the risks posed by supplement contamination, many athletes, practitioners and researchers espouse a “food-first” approach to sports nutrition. As honey is a natural substance comprised of ~80% carbohydrate (primarily fructose and glucose), and is known to possess antioxidant, antimicrobial, and anti-inflammatory properties, there exists a theoretical basis for its use as a nutritional supplement in exercising populations. This review summarised the available literature which has investigated the effects of honey supplementation when combined with exercise over a number of different time-frames, dosages, and modalities. Whilst the large methodological differences within the studies represented a substantial limitation, information is presented which may inform worthwhile future research.

Compared with other forms of carbohydrate, honey ingestion has had a similar effect on exercise performance, perceptions of fatigue, blood glucose concentrations, and immunological responses when consumed immediately prior to and/or during exercise, although some positive influence has been observed. When routinely consumed over multiple weeks, honey may attenuate many of the immune perturbations typically associated with a programme of moderate-to-intense exercise. Unfortunately, the research designs employed and the level of detail with which the methods have been reported make it difficult to establish whether the observed responses are attributable to the intrinsic properties of honey itself, or reflect other factors such as discrepancies in carbohydrate intake between conditions. Similarly, the same limitations mean that honey may have had certain effects (either positive or negative) which could have been masked by “noise” from external influences. Despite the lack of conclusive evidence, theory supports the use of honey, particularly as a potential ergogenic aid when consumed around exercise. Future research should take a robust approach to assessing whether honey may offer benefits to physical, skilled, or cognitive performance during different modalities, durations, and intensities of exercise, when directly compared to equivalent volumes of carbohydrates delivered in traditional forms. From these authors’ perspective, the application to skilled performance during team sports may be of particular interest. Moreover, studies directly comparing the responses of male and female athletes would be a valuable addition to the knowledge base.

## Figures and Tables

**Table 1:** Studies examining the effect of acute honey supplementation on biochemical responses to a single exercise session.

Study	Participants	Design	Exercise Stimulus	Nutritional Intervention	Data Collection Method and Time-Points	Outcome Variables	Main Results
**Abbey and Rankin** [47]	Male soccer players of NCAA Division I, post collegiate, and club standard (*n* = 10).	Randomised, single blind, crossover.	Soccer specific exercise (5 × 15 min blocks, plus 10 min half-time), followed by progressive 20 m shuttle run to fatigue.	6% CHO-electrolyte solution (honey, commercial sports drink, or placebo). 8.8 mL∙kg^−1^ (0.5 g∙kg∙body mass^−1^) CHO consumed 30 min pre-exercise and at half-time.	Blood samples at 30 min pre-exercise (T1), immediately post-exercise (T2), 60 min post-exercise (T3).	Blood glucose, insulin, cortisol, plasma volume, IL-1ra, IL-6 and IL-10, total ORAC, and plasma ORAC.	Plasma IL-1ra was ↓ at T2 for honey vs. sports drink, and ↓ at T3 for honey vs. placebo. ↔ between trials for glucose, insulin, cortisol, plasma volume, IL-6, IL-10, total or plasma ORAC.
**Ahmad et al.** [48]	Male runners of recreational standard (*n* = 10).	Randomised, single blind, crossover.	60 min run at ~65% V̇O_2_ max, followed by 2 h ‘rehydration phase’ and 20 min treadmill running test.	6.8% CHO solution (honey) or water to recover 150% of body mass lost during run one. Fluid consumed 0 min (60% of mass loss), 30 min (50%), and 60 min (40%) after run one.	Blood samples at pre (T1), immediately post (T2), 30 min post (T3), 60 min post (T4), 90 min post (T5), and 120 min post (T6) 60 min run, and immediately post 20 min run (T7).	Blood glucose, serum insulin, haematocrit, and serum osmolality.	Serum insulin was ↑ at T3-T6 for honey vs. water. Serum osmolality was ↑ at T4 for honey vs. water. ↔ between trials for glucose or haematocrit.
**Earnest et al.** [46]	Amateur male cyclists (*n* = 9).	Randomised, double blind, counterbalanced, crossover.	64 km time trial on cycling ergometer.	15 g of gel (honey, dextrose, or placebo) with 250 mL H_2_O consumed every 16 km (5 × 15 g total) plus an additional 250 mL of water every 3.2 km.	Blood samples at pre-exercise (T1), 16 km (T2), 32 km (T3), 48 km (T4), and 64 km (T5).	Blood glucose and insulin concentrations.	↔ between trials for glucose or insulin. In dextrose, glucose at T4 was ↓ vs. T1 (not the case for honey or placebo).
**Kreider et al.** [49]	Resistance trained individuals (*n* = 40; males: *n* = 19, females: *n* = 21).	Randomised, four independent groups.	3 sets of 10 repetitions at approximately 70% of 1RM on chest press, seated row, shoulder press, lat pulldown, leg extension, leg curl, biceps curl, triceps extension, and leg press.	40 g of whey protein with 120 g of either sucrose, powdered honey, or maltodextrin consumed within 5 min post-exercise. Other group consumed no supplement.	Blood samples at pre-exercise (T1), post exercise (T2), and 30 min (T3), 60 min (T4), 90 min (T5), and 120 min (T6) post-feeding.	Glucose, insulin, testosterone, cortisol, testosterone: cortisol ratio, WBC, neutrophils, total neutrophils: total lymphocytes ratio, creatinine, BUN, BUN: creatinine ratio, CK, LDH, AST, and ALT.	Glucose at T4 was ↑ for honey vs sucrose, and at T3 was ↑ for honey vs. sucrose, maltodextrin, and no CHO. Insulin at T3-T6 was ↑ for honey, sucrose, and maltodextrin vs. no CHO. ↔ between groups for testosterone, cortisol, testosterone:cortisol, WBC, neutrophils, total neutrophils, total lymphocytes, LDH, AST, or ALT. BUN: creatinine at T5, was ↑ for honey and maltodextrin, and at T6 was ↑ for honey and sucrose vs. no CHO.
**Łagowska et al.** [45]	Trained male rowers (*n* = 11).	Randomised, crossover.	Rowing ergometer: 2 × 40 min with a 5 min break, performed at an intensity corresponding to ~75% of the onset of blood lactate accumulation.	150 mL of CHO solution (either commercial sports drink; 7.8% CHO, or ‘natural’ drink containing banana, fruit juice, and honey; 6.7% CHO), consumed immediately pre-exercise and every 15 min during exercise (6 × 150 mL total).	Blood samples at pre-exercise (T1), and 3 min post-exercise (T2).	Blood glucose, lactate, chemical antioxidants, urea, CK, haematocrit, leukocytes, WBC, lymphocytes, monocytes, and granulocytes.	Glucose was ↓ at T2, for natural vs. commercial drink. Glucose at T2, was ↓ for natural, but ↑ for commercial drink vs. T1. Chemical antioxidant level at T2, was ↓ for natural vs. commercial drink. ↔ between trials for lactate, urea, CK, haematocrit, leukocytes, WBC, lymphocytes, monocytes, or granulocytes.

ALT: alanine aminotransaminase, AST: aspartate aminotransaminase, BUN: blood urea nitrogen, CHO: carbohydrate, CK: creatine kinase, IL-1ra: interleukin-1 receptor antagonist, IL-6: interleukin-6, IL-10: interleukin-10, LDH: lactate dehydrogenase, NCAA: National Collegiate Athletic Association, ORAC: oxygen radical absorbance capacity, WBC: white blood cell counts, 1RM: one repetition maximum, ↑: increased/higher, ↓: decreased/lower, ↔: no difference.

**Table 2:** Studies examining the effect of honey supplementation over the course of multiple weeks on biochemical responses to exercise.

Study	Participants	Design	Exercise Stimulus	Nutritional Intervention	Data Collection Method and Time-Points	Outcome Variables	Main Results
**Gmunder et al.** [52]	Long distance runners (*n* = 16: male: *n* = 13, female: *n* = 3). Standard not specified.	Randomised, double blind, two independent groups.	27 day training period, followed by 21 km running time trial.	3 x 10 mL per day (30 mL per day for 31 days) of either a supplement comprised of herbal yeast, malt, honey, and orange juice, or a supplement comprised of sucrose and caramel.	Blood samples at day 0 (T1), pre 21 km run at day 27 (T2), immediately post run (T3), two days post run (T4).	WBC, leukocyte counts, lymphocyte counts, Con A, CD3, CD4, CD16, CD16/8, CD19, IgA, IgE, IgM, IgG subclasses 1-4, β2M, IL-2 receptors, neopterin, plasma proteins, cortisol, adrenaline, noradrenaline.	↔ between groups for any blood marker. In honey, natural killer/suppressor cells (CD8, CD16, and CD16/8), and IgG subclass 1 at T4 were ↓, whilst Con A, and IgG subclass 2 at T4 were ↑ vs. T3 (not the case for sucrose). In sucrose, neopterin and β2M at T4 were ↓ vs. T3 (not the case for honey).
**Hajizadeh et al.** [51]	Amateur male cyclists (*n* = 24).	Randomised, two independent groups.	16 week training period.	70 g honey dissolved in 250 mL distilled water. Consumed 90 min prior to each training session for 16 weeks. Other group consumed no supplement.	Blood samples at week 0 (T1), immediately (T2), 12 h (T3), and 24 h (T4) after the last training session in week 8, and immediately (T5), 12 h (T6), 24 h (T7), 7 days (T8), and 30 days (T9) after the last training session in week 16.	Lymphocyte counts, DNA damage, IL-1β, IL-6, IL-8, IL-10, TNF-a, glutathione, TAS, hydrogen peroxide, and lipid peroxide.	Hydrogen peroxide, and lipid peroxide, IL-1β, IL-6, IL-10, and TNF-a at T2-T8, were ↓ for honey vs. no supplement. Glutathione at T4-T7 was ↑ for honey vs. no supplement. T2-T7 was ↑ for honey vs. no supplement.
**Ooi et al.** [53]	Females (*n* = 37).	Four (matched) independent groups.	Two groups: 3 aerobic dance sessions per week for 6 weeks. Other two groups: no exercise.	Two groups: 20 g honey with 300 mL water consumed daily for 6 weeks. Other two groups: no supplementation.	Blood samples at week 0 (T1) and after week 6 (T2).	ALP and 1CTP.	ALP at T2 for honey, and honey plus exercise was ↑ vs. T1 (not the case with no honey). ↔ between groups for ALP or 1CTP. ↔ between T1 and T2 for 1CTP.
**Tartiban and Maleki** [50]	Male amateur road cyclists (*n* = 39).	Randomised, double blind, two independent groups.	8 weeks intensified training period.	70 g of honey or 70 g CHO-free sweetener, with 250 mL water. Consumed 90 min prior to each training session for 8 weeks.	Semen samples at week 0 (T1), immediately (T2), 12 h (T3), and 24 h (T4) after the last training session in week 4, and immediately (T5), 12 h (T6), and 24 h (T7) after the last training session in week 8.	Semen volume, motility, morphology, concentration, and number of spermatozoa, plus IL-1β, IL-6, IL-8, TNF-α, ROS, malondialdehyde, and antioxidants superoxide dismutase, catalase, and TAC.	Semen volume at T6 was ↑ for honey vs. placebo. Semen motility, morphology and TAC at T6-T7, semen concentration at T4-T5, number of spermatozoa at T2-T3 and T5-T7, catalase at T4 and T5-T7, and superoxide dismutase at T2-T3 and T5-T7 were all ↑ for honey vs. placebo. IL-1β at T2 and T5-T7, IL-6 at It2-T7, IL-8 at T2-T3, TNF-α at T4, ROS at T4-T7, and malondialdehyde at T5-T7 were all ↓ for honey vs. placebo.

ALP: serum alkaline phosphatase, β2m: beta-2 microglobulin, CD3: cluster of differentiation 3, CD4: cluster of differentiation 4, CD16: cluster of differentiation 16, CD16/8: cluster of differentiation 16/8, CD19: cluster of differentiation 19, CHO: carbohydrate, Con A: concanavalin A, IgA: immunoglobulin A, IgE: immunoglobulin E, IgM: immunoglobulin M, IgG immunoglobulin G, IL-1β: interleukin-1 beta, IL1ra: interleukin-1 receptor antagonist, IL-2: interleukin 2, IL-6: interleukin-6, IL-8: interleukin-8, IL-10: interleukin-10, LDH: lactate dehydrogenase, ORAC: oxygen radical absorbance capacity, ROS: reactive oxygen species, TAC: total antioxidant capacity, TAS: total antioxidant status, TNF-α: tumor necrosis factor alpha, WBC: white blood cell counts, 1CTP: serum C-terminal telopeptide of type 1 collagen, ↑: increased/higher, ↓: decreased/lower, ↔: no difference.

**Table 3:** Studies examining the effect of honey supplementation on exercise (physical or skilled) performance.

Study	Participants	Design	Exercise Stimulus	Nutritional Intervention	Data Collection Method and Time-Points	Outcome Variables	Main results
**Abbey and Rankin** [47]	Male soccer players of NCAA Division I, post collegiate, and club standard (*n* = 10).	Randomised, single blind, crossover.	Soccer specific exercise (5 × 15 min blocks, plus 10 min half-time), followed by progressive 20 m shuttle run to fatigue.	6% CHO-electrolyte solution (either honey, commercial sports drink, or placebo). 8.8 mL∙kg^−1^ (0.5 g∙kg∙body mass^−1^) CHO consumed 30 min pre-exercise and at half-time.	220 m running time trial, dribbling/agility assessment, and soccer shooting test all performed every 15 min during exercise: 220 m running time trial Progressive 20 m shuttle run to fatigue performed following 75 min of exercise.	Time taken to complete (time trial, and dribbling/ agility test), number of targets hit (shooting assessment). Time to exhaustion.	↔ between trials for any performance measure.
**Ahmad et al.** [48]	Male runners of recreational standard (*n* = 10).	Randomised, single blind, crossover.	60 min run at ~65% V̇O_2_ max, followed by 2 h ‘rehydration phase’ and 20 min treadmill running test.	Either 6.8% CHO solution (honey) or water, to recover 150% of body mass lost during run one consumed at 0 min (60% of mass loss), 30 min (50%), and 60 min (40%) after run one.	20 min treadmill running test performed 120 min following completion of 60 min run	Total distance covered.	20 min running performance was ↑ for honey vs. water.
**Earnest et al.** [46]	Amateur male cyclists (*n* = 9).	Randomised, double blind, counterbalanced, crossover.	64 km time trial on cycling ergometer.	15 g of gel (honey, dextrose, or placebo) with 250 mL water consumed every 16 km (5 × 15 g total). Plus an additional 250 mL of water every 3.2 km.	64 km cycling ergometer test.	Time taken to complete 64 km, and per 16 km. Mean power over 64 km, and per 16 km.	↔ between trials for time taken to complete, or mean power over 64 km. In honey and dextrose trials, mean power over 48-64 km was ↑ vs. 0-16 km, 16-32 km, and 32-48 km (not the case for placebo). In placebo, time taken for 48-64 km and 32-48 km was ↑ vs. 0-16 km, (not the case for honey or dextrose).
**Hajizadeh et al.** [51]	Amateur male road cyclists (*n* = 24).	Randomised, two independent groups.	16 week training period.	70 g honey dissolved in 250 mL distilled water. Consumed 90 min prior to each training session for 16 weeks. Other group consumed no supplement.	5 km, and 40 km cycling ergometer tests, and power assessment at week 0 (T1) and week 16 (T2).	Time taken to complete (5 km and 40 km tests), peak power output (power assessment).	↔ between groups for time taken to complete 5 km or 40 km, or peak power output at T2. For all measures, performance in both groups was ↑ at T2 vs. T1

CHO: carbohydrate, NCAA: National Collegiate Athletic Association, ↑: increased/higher, ↓: decreased/lower, ↔: no difference.

**Table 4:** Studies examining the effect of honey supplementation on perceptual responses around exercise.

Study	Participants	Design	Exercise Stimulus	Nutritional Intervention	Data collection Method and Time-Points	Outcome Variables	Main Results
**Abbey and Rankin** [47]	Male soccer players of NCAA Division I, post-collegiate, and club standard (*n* = 10).	Randomised, single blind crossover.	Soccer-specific exercise (5 × 15 min blocks, plus 10 min half-time), followed by progressive 20 m shuttle run to fatigue.	6% CHO-electrolyte solution (honey, commercial sports drink, or placebo). 8.8 mL∙kg^−1^ (0.5 g∙kg∙body mass^−1^) CHO consumed 30 min pre-exercise and at half-time.	Every 15 min during exercise, scale not reported.	RPE.	↔ between trials for RPE.
**Ahmad et al.** [48]	Male runners of recreational standard (*n* = 10).	Randomised, single blind crossover.	60 min run at ~65% V̇O_2_ max, followed by 2 h ‘rehydration phase’ and 20 min treadmill running test on.	6.8% CHO solution (honey) or H_2_O to recover 150% of body mass lost during run one. Fluid consumed 0 min (60% of mass loss), 30 min (50%), and 60 min (40%) after run one.	Likert scale (1–5), 0 min (T1), 30 min (T2), and 60 min (T3) after run one.	Perceptions of thirst, sweetness, nausea, fullness and stomach upset.	Perceptions of sweetness at T1-T3 were ↑ for honey vs. for honey vs. water. ↔ between trials for perceptions of thirst nausea, fullness, or stomach upset between trials.
**Earnest et al.** [46]	Amateur male cyclists (*n* = 9).	Randomised, double blind, counterbalanced, crossover.	64 km time trial on cycling ergometer.	15 g of gel (honey, dextrose, or placebo) with 250 mL water consumed every 16 km. Plus an additional 250 mL of water every 3.2 km.	Likert scale (6–20) at pre-exercise, 16 km, 32 km, 48 km, and 64 km.	RPE.	↔ between trials for RPE.
**Kreider et al.** [49]	Resistance trained individuals (*n* = 40; males: *n* = 19, females: *n* = 21).	Randomised, four independent groups.	3 sets of 10 repetitions at approximately 70% of 1 RM on chest press, seated row, shoulder press, lat pulldown, leg extension, leg curl, biceps curl, triceps extension, and leg press.	40 g of whey protein with 120 g of either sucrose, powdered honey, or maltodextrin. Consumed within 5 min post-exercise. Other group consumed no supplement.	Likert scale (1–10) at pre-exercise (T1), post exercise (T2), and 30 min (T3), 60 min (t4), 90 min (T5), and 120 min (T6) post-feeding.	Severity of perceived hypoglycaemia, dizziness, fatigue, headache, and stomach upset.	↔ between groups for perceptions of hypoglycaemia, dizziness, fatigue, headache, or stomach upset.
**Łagowska et al.** [45]	Trained male rowers (*n* = 11).	Randomised, crossover.	Rowing ergometer: 2 × 40 min with a 5 min break, performed at an intensity corresponding to ~75% of the onset of blood lactate accumulation.	150 mL of CHO solution (either commercial sports drink; 7.8% CHO, or ‘natural’ drink containing banana, fruit juice, and honey; 6.7% CHO), consumed immediately pre-exercise and every 15 min during exercise (6 × 150 mL total).	Likert scale (1–5) at post-exercise:	Perceptions of taste, smell, thirst quenching ability, beverage consistency, and refreshment.	Satisfaction with beverage consistency was ↓ for natural vs. commercial drink. ↔ between trials for perceptions of taste, smell, thirst quenching ability, or refreshment.

CHO: carbohydrate, NCAA: National Collegiate Athletic Association, RPE: rating of perceived exertion, 1RM: one repetition maximum, ↑: increased/higher, ↓: decreased/lower, ↔: no difference.
